# The Analysis of the Correlations Between Bullying Perpetration, Victimization, and Assertive Behavior Among Primary School Students in Romania

**DOI:** 10.3390/children13060724

**Published:** 2026-05-23

**Authors:** Andreea Maria Sticea, Bogdan Constantin Ungurean, Bogdan Constantin Rață, Adrian Cojocariu

**Affiliations:** 1 Doctoral School in Sports and Physical Education Science, Faculty of Physical Education and Sports, “Alexandru Ioan Cuza” University of Iasi, 507184 Iasi, Romania; sticea_andreea@yahoo.com; 2 Faculty of Physical Education and Sports, “Alexandru Ioan Cuza” University of Iasi, 507184 Iasi, Romania; cadriano@uaic.ro; 3 Department of Physical Education and Sports Performance, Faculty of Movement, Sports and Health Sciences, “Vasile Alecsandri” University of Bacău, 600115 Bacau, Romania; rata.bogdan@ub.ro

**Keywords:** bullying perpetration, victimization, assertive behavior, primary school students

## Abstract

**Background/Objectives:** The phenomenon of bullying represents a major issue in school settings worldwide, despite extensive research on the topic. This study aimed to investigate the associations between bullying perpetration, victimization, and assertive behavior among primary school students. **Methods:** The sample consisted of 352 fourth-grade students (174 boys and 178 girls; aged 10–11 years) from 7 schools in Iași, Romania. Bullying perpetration and victimization were assessed using the Adolescent Peer Relations Instrument–Bully/Target (APRI-BT), while assertive behavior was assessed using the Children’s Assertive Behavior Scale (CABS). Statistical analyses were performed using IBM SPSS Statistics version 20. **Results:** Positive correlations ranging from weak to strong were identified between bullying perpetration and victimization in both boys and girls. Bullying perpetration and victimization were also associated with assertive behavior and nonassertive behaviors, although most correlations were weak in magnitude. **Conclusions**: The findings highlight the complexity of bullying-related interpersonal dynamics during late childhood and may have implications for school-based social and emotional learning and bullying prevention interventions during this developmental stage.

## 1. Introduction

Various forms of victimization may occur in school settings, most frequently between peers, but they may also involve teachers or other members of the school staff [1,2]. Aggression represents the general category, while violence and bullying are subcategories of aggressive behavior [3]. School violence encompasses multiple forms of violence, including physical, psychological, and sexual violence, and also includes school bullying as one of its most common manifestations [1,2]. School bullying is distinguished from other types of aggressive behavior by its deliberate nature, the recurrence of behaviors, and the power imbalance between the aggressor and the victim. Bullying can be manifested by one or more individuals and results in negative physical, psychological, social, and/or educational effects [4,5]. Bullying can take direct and indirect forms. Direct (physical) bullying involves observable behaviors such as hitting, pushing, or damaging personal belongings. Indirect (psychological) bullying includes verbal forms such as teasing, name-calling, and spreading rumors, as well as nonverbal forms such as threatening gestures and social exclusion [6]. School bullying is conceptualized as a triadic social process involving perpetrators, victims, and bystanders. Perpetrators initiate bullying behaviors, while victims are the recipients of these behaviors. A distinct group is represented by bully–victims, characterized by a dual behavioral pattern [7]. Bystanders, who witness bullying episodes, may assume different roles: assistants (who actively participate in bullying), reinforcers (who indirectly encourage the behavior, for example, by laughing), outsiders (who remain passive), and defenders (who support victims and report incidents) [8,9]. Thus, bullying can be understood as a social process maintained by bystanders, who contribute to reinforcing the perpetrators’ status within the peer group [10]. Increased access to and use of technology among children and adolescents has led to the emergence of a new form of aggression, namely cyberbullying. On the one hand, there is evidence suggesting that traditional bullying and cyberbullying share common characteristics. On the other hand, cyberbullying also presents specific features related to its digital nature. The literature does not provide a clear consensus on whether cyberbullying should be conceptualized as a distinct phenomenon or as an extension of traditional bullying. Nevertheless, cyberbullying is often examined as a separate construct [7,11,12].

Given its prevalence and impact, school bullying has received considerable attention in the international scientific literature. A meta-analysis including 80 international studies found that the prevalence of bullying victimization is 36%, while the prevalence of bullying perpetration is 35% [13]. Research on school bullying in Romania is limited compared to the international literature. Available data indicate that approximately 33.8% of students have reported being victimized, a rate within the range reported in other European countries [2]. Evidence also suggests gender differences, with a higher prevalence of bullying perpetration among boys (30.8%) compared to girls (20.6%), as well as higher victimization rates among boys (19.8%) than girls (13.9%) [14]. Studies conducted in Romania also suggest mixed findings regarding gender differences in bullying involvement. In a sample of students aged 9–11 years, boys were found to perpetrate bullying more frequently than girls [15]. In contrast, in a sample of students aged 10–14 years, girls were reported to be more likely to perpetrate bullying than boys [16]. Regarding victimization, no significant gender differences were identified [15,16]. These mixed findings may be explained by differences in the age range of the samples, as forms of bullying can change across developmental stages. They may also reflect methodological differences across studies. Researchers’ interest in this social issue is driven by the negative consequences of bullying for those involved. Students may experience difficulties in concentration [17], poorer academic performance and absenteeism [18], as well as depression and suicidal ideation during school years [19] and later in adulthood [20]. In addition, school bullying has been associated with substance use [21] and risky or illegal behaviors in adulthood [22].

As bullying represents a form of social interaction, the social skills of individuals involved in these interactions play an important role in understanding its dynamics [23]. Social skills are acquired behaviors that allow individuals to engage in effective and socially appropriate interactions with others [24]. Research on the relationship between social skills and bullying indicates that individuals who possess more developed or effective social skills tend to be involved in bullying less frequently [25,26]. In contrast, lower levels of social skills have been associated with increased participation in bullying [25]. Moreover, deficits in social skills may increase the likelihood of victimization. Evidence also suggests that well-developed social skills may serve as a protective factor against bullying, emphasizing the importance of assertive behavior in enabling individuals to express their thoughts and emotions in a clear and respectful manner [27,28].

Assertiveness represents an individual’s ability to express ideas, feelings, and boundaries while respecting others, maintaining positive relationships, and considering the potential consequences of their actions [29]. The relationships between bullying perpetration, victimization, and assertive behavior can be further understood through the social information processing theory [30], which explains how individuals interpret social cues and generate behavioral responses in interpersonal situations. Studies suggest that perpetrators and victims may exhibit different patterns of social information processing. In the case of perpetrators, some studies indicate a lack of significant biases in interpreting others’ intentions, whereas others suggest the presence of hostile attribution biases, strategic goal selection, and a perceived efficacy of aggressive behavior, potentially driven by the pursuit of social dominance and status within the peer group [31,32,33,34,35]. In contrast, victims tend to exhibit heightened sensitivity to threatening social cues, biased interpretations of social situations, and a tendency to generate less assertive and more avoidant or reactive responses [36,37]. These characteristics may contribute to increased vulnerability to victimization and reduced effectiveness in managing social interactions.

Studies examining the associations between bullying perpetration, victimization, and assertive behavior are limited and have reported inconsistent findings. While some studies indicate that higher levels of assertive behavior are associated with increased bullying perpetration and lower levels of victimization [38], other findings show negative or non-significant associations between assertive behavior and both bullying perpetration and victimization [39,40]. When expressed in an adaptive manner, assertive behavior may facilitate setting boundaries and reduce experiences of victimization. In the case of perpetrators, behaviors interpreted as assertive may in fact reflect underlying dominant or aggressive tendencies. These mixed results can be explained by methodological and age differences across studies.

Starting from around the age of 10, children begin to experience substantial developmental changes across physical, cognitive, social, and emotional domains [41]. Peer relationships become progressively more influential, contributing to the development of social behavior [42]. In this context, children in Grade 4 constitute a relevant group for examining assertive behavior in relation to bullying perpetration and victimization, as this social skill plays an increasingly important role in peer interactions.

## 2. Materials and Methods

### 2.1. Aim and Hypotheses

The aim of this study is to examine the associations between bullying perpetration, victimization and assertive behavior among primary school students in Romania.

**Hypothesis** **1.**  *Bullying perpetration will be positively associated with victimization among primary school students in Romania*.

**Hypothesis** **2.**  *Bullying perpetration will be associated with assertive behavior among primary school students in Romania*.

**Hypothesis** **3.**  *Victimization will be associated with assertive behavior among primary school students in Romania*.

### 2.2. Research Subjects

A total of 352 fourth-grade students (174 boys and 178 girls; aged 10–11 years) from seven schools in Iași, Romania, participated in this study. Participants were selected using convenience sampling.

### 2.3. Procedure

Students were informed about the aim of the study using language appropriate to their age. Participation was entirely voluntary, and no reward was provided to the children, their families, or the school. The protocol consisted of two questionnaires administered in this order: one assessing students’ involvement in bullying, both as perpetrators and victims, and another assessing assertive behavior. Before completing the questionnaires, students were given the opportunity to become familiar with the researcher. Data were collected anonymously by one of the researchers. All questionnaires were completed during the same session and lasted approximately one hour for each class. The questionnaires were administered between 11 November 2024 and 17 December 2024.

### 2.4. Ethical Considerations

The study was conducted in accordance with the Declaration of Helsinki. The study protocol received approval from the Research Ethics Committee of “Alexandru Ioan Cuza” University of Iași, Faculty of Physical Education and Sport (protocol code: 33; date of approval: 4 November 2024). Following approval from the school principals, informed consent was obtained from the parents or guardians of the participating children. Only students whose parents provided consent were included in the study. Children provided their assent to participate after being informed about the purpose of the research. Students were informed that they could withdraw from the study at any time without any consequences. A teacher was present throughout the data collection process and was available to provide support and assistance if needed.

### 2.5. Instruments and Measures

Children’s involvement in bullying perpetration and victimization was assessed using a Romanian version of the Adolescent Peer Relations Instrument–Bully/Target (APRI-BT) [43]. The APRI-BT is a 36-item self-report questionnaire designed to evaluate bullying perpetration and victimization across three dimensions: physical, verbal, and social. In the bullying section, participants report the frequency with which they engaged in various bullying behaviors toward peers during the previous school year (example item: “Got into a physical fight with a student because I didn’t like them”). In the victimization section, participants indicate how often they experienced these behaviors from other peers (example item: “I was called names I didn’t like”). Responses are provided on a six-point Likert scale (1 = Never, 2 = Sometimes, 3 = Once or twice a month, 4 = Once a week, 5 = Several times a week, 6 = Daily). Lower scores indicate lower involvement in bullying perpetration or victimization, whereas higher scores reflect more frequent involvement. Total scores for both bullying perpetration and victimization range from 18 to 108, with higher scores indicating higher levels of bullying perpetration or victimization. The Romanian adaptation of the APRI-BT demonstrated good psychometric properties, including confirmation of the factorial structure and measurement invariance across gender, age, and clinical status [44].

Assertive behavior was assessed using the Children’s Assertive Behavior Scale (CABS) [45]. The instrument evaluates children’s social behavior across a variety of interpersonal situations, examining assertive, passive, and aggressive responses. The situations assessed involve different social skills, such as giving and receiving compliments, initiating and ending conversations, requesting favors, responding to insults, and expressing positive or negative emotions. The scale contains 27 items, each including 5 responses positioned along a continuum ranging from very passive to very aggressive behavior. Participants are asked to choose the response that best reflects their usual reaction in each situation. Assertive responses involve expressing thoughts, feelings, and personal rights in an appropriate manner while respecting others. Passive responses reflect avoidance, inhibition, or failure to address the situation directly. Aggressive responses involve hostile interaction patterns, including verbal or physical aggression and threats. Response options are scored as follows: −2 = very passive, −1 = partially passive, 0 = assertive, +1 = partially aggressive, and +2 = very aggressive. The scoring procedure provides three indices: an assertiveness score based on the sum of all responses, a passive behavior score derived from negative responses, and an aggressive behavior score derived from positive responses. The passive and aggressive scores reflect different forms of nonassertive responding. As CABS has not been validated for the Romanian school population, a preliminary cross-cultural adaptation process was conducted. The instrument was translated from English into Romanian by a psychotherapist accredited by the Romanian College of Psychologists, who was familiar with both cultures. The translation was reviewed by three researchers, after which the Romanian version was back-translated into English by another researcher. The back-translated version was subsequently compared with the original English questionnaire for accuracy.

### 2.6. Statistical Analyses

The obtained data were analyzed using IBM SPSS Statistics version 20 (IBM Corp, Armonk, NY, USA). The Kolmogorov–Smirnov test was used to assess the normality of data distribution. Cronbach’s Alpha coefficient, Corrected Item-Total Correlation, and Cronbach’s Alpha if Item Deleted were calculated to evaluate the internal consistency of the CABS. Spearman’s correlation analysis was performed to examine the associations between bullying perpetration, victimization, and assertive behavior.

## 3. Results

The results of the Kolmogorov–Smirnov test indicated that the distribution of data for assertive, passive, and aggressive behaviors differed significantly from a normal distribution (*p* < 0.001) in both boys and girls. The results of the Kolmogorov–Smirnov test also indicated significant deviations from normality (*p* < 0.001) for all bullying-related variables in both groups, boys and girls. Therefore, nonparametric tests were used to analyze the correlations (see Table 1).

According to the data presented in Table 2, the Corrected Item-Total Correlation of the scale items ranged between 0.049 and 0.417. All items demonstrated positive correlations with the total scale score, indicating consistency with the overall construct measured by the scale. The Cronbach’s Alpha if Item Deleted values ranged between 0.703 and 0.732, while the overall Cronbach’s Alpha coefficient of the scale was 0.725, demonstrating acceptable internal consistency. Although several items showed lower correlations, deleting individual items did not substantially improve the overall internal consistency coefficient, which remained acceptable.

Table 3 presents the results of Spearman correlations between bullying perpetration, victimization, and assertive, passive, and aggressive behaviors among boys.

The results revealed statistically significant positive correlations between all forms of bullying perpetration and victimization, with correlation coefficients ranging from weak to moderate (*p* < 0.01).

The analysis of the correlations between bullying perpetration and assertive, passive, and aggressive behaviors revealed several statistically significant associations of weak magnitude (*p* < 0.01; *p* < 0.05). Assertive and aggressive behaviors were positively associated with bullying perpetration, particularly with total, verbal, and social bullying. In contrast, passive behavior showed a weak negative association with social bullying.

Regarding the correlations between victimization and assertive, passive, and aggressive behaviors, several statistically significant associations of weak magnitude were identified (*p* < 0.01; *p* < 0.05). Assertive and aggressive behaviors were positively associated with victimization, particularly with total, physical, verbal, and social victimization. Passive behavior showed weak negative associations with total, physical, and social victimization.

Table 4 presents the results of Spearman correlations between bullying perpetration, victimization, and assertive, passive, and aggressive behaviors among girls.

The results revealed statistically significant positive correlations between all forms of bullying perpetration and victimization, with correlation coefficients ranging from weak to strong (*p* < 0.01; *p* < 0.05).

The analysis of the correlations between bullying perpetration and assertive, passive, and aggressive behaviors revealed several statistically significant associations ranging from weak to moderate in magnitude (*p* < 0.01; *p* < 0.05). Assertive and aggressive behaviors were positively associated with all forms of bullying perpetration. In contrast, passive behavior was negatively associated with total and social bullying.

Regarding the correlations between victimization and assertive, passive and aggressive behaviors, several statistically significant correlations of weak magnitude were identified (*p* < 0.01; *p* < 0.05). Assertive and aggressive behaviors were positively associated with victimization, particularly with physical and social victimization. Passive behavior showed negative associations with physical and social victimization.

## 4. Discussion

This study aimed to explore the correlations between bullying perpetration, victimization, and assertive behavior among primary school students.

The internal consistency of the CABS was acceptable and comparable to that reported in the original study [45], providing preliminary support for the use of the instrument in the present study, although additional psychometric validation remains necessary for the Romanian school population.

The results of our study revealed positive associations between bullying perpetration and victimization in both boys and girls. Similar findings have been reported in previous studies [46,47,48]. These results may reflect the complex nature of bullying dynamics, in which some children may simultaneously experience victimization and perpetrate bullying across peer interactions.

The present study identified positive associations between bullying perpetration and assertive behavior in both boys and girls, although these correlations appeared more consistent among girls. Similar findings have been reported in previous studies involving lower secondary school students. One possible explanation is that some behaviors perceived as assertive may be used instrumentally within peer interactions, for purposes that are harmful toward victims [38].

Certain bullying perpetration variables showed inverse associations with passive behavior in both boys and girls. In contrast to our findings, another study involving students aged 10–12 years reported no significant correlations between bullying perpetration and passive behavior [40]. Passive behavior may reflect avoidance of confrontational peer interactions and lower involvement in socially dominant behaviors. Given that bullying perpetration is generally characterized by dominance-oriented interactions, children with passive behavior may be less likely to engage in bullying perpetration.

Several bullying perpetration variables were positively associated with aggressive behavior, particularly among girls. Similar findings have been reported in studies identifying positive correlations between bullying perpetration and aggressive, authoritarian, proactive, or reactive behaviors among students of similar ages [40,49,50]. These associations between aggressive behavior and bullying perpetration may suggest difficulties in social, emotional, and cognitive functioning, which may be expressed through aggressive and antisocial responses during peer interactions, including bullying perpetration. However, these comparisons should be interpreted cautiously, as previous studies employed instruments specifically designed to assess these forms of aggression or social skills, unlike our study, which used a measure of assertive behavior along with tendencies toward passive and aggressive behavior. Consequently, the constructs assessed across studies are not equivalent.

Our study also yielded several noteworthy findings. Weak positive associations were identified between certain forms of victimization and assertive behavior in both boys and girls. This is a counterintuitive finding. Assertive behavior has been associated in some studies with lower levels of victimization [38,39]. Findings partially similar to ours were also reported in another study involving students of similar ages, although only among boys, but the scale used in this study assessed social skills [40]. One possible explanation is that, at this age, children may be more likely to express disagreement and defend personal boundaries. Within bullying interactions, such responses may generate negative reactions from perpetrators, potentially increasing exposure to certain forms of victimization, depending on gender.

The results also showed weak negative associations between certain victimization variables and passive behavior in both boys and girls. Previous studies have reported positive correlations between victimization and passive behavior among girls [40], which is inconsistent with our findings, probably due to differences in the instruments used across studies. Passive behavior may reflect avoidance and lower visibility within peer-group dynamics, potentially reducing exposure to certain forms of victimization.

Weak positive associations with aggressive behavior were observed across certain victimization variables in boys and girls. Similar results have been reported in studies that identified correlations between victimization and aggressive, authoritarian or reactive behaviors among children of similar ages [40,49,50]. Our results may suggest that children involved in victimization may adopt reactive behaviors as maladaptive strategies for coping with these negative peer experiences. However, these results should be viewed with caution, as the instruments used across studies were not fully comparable in terms of the behavioral constructs assessed.

The present study contributes to both the Romanian and the international literature by exploring the correlations between bullying perpetration, victimization, and assertive behavior among primary school students. The study also contributes to the preliminary exploration of the Romanian adaptation of the CABS in a school population. The findings highlight the dynamics of bullying among children aged 10–11 years, an important developmental stage characterized by cognitive, social, and emotional changes.

Our findings may have practical implications for school-based social and emotional learning and bullying prevention programs. The identified correlations between bullying perpetration, victimization, and assertive behavior may suggest the importance of interventions targeting emotional regulation, assertive behavior, conflict management, and prosocial peer interactions during late childhood. Such educational programs may contribute to reducing the bullying phenomenon before adolescence.

Several limitations of the present study should be acknowledged. The study used a preliminary Romanian adaptation of the CABS, for which additional psychometric validation is still necessary. The cross-sectional design does not allow conclusions regarding causal relationships between bullying perpetration, victimization, and assertive behavior. Another limitation concerns the exclusive use of self-report questionnaires. Responses may have been influenced by potential difficulties in understanding some questionnaire items, by the tendency to provide socially desirable answers, or by limitations related to recalling previous experiences, potentially affecting the identified correlations. Finally, the use of convenience sampling limits the generalizability of the results to broader populations of Romanian students.

Future research should conduct additional psychometric validation of the Romanian version of the CABS, including factorial and construct validity analyses. Longitudinal and multi-informant studies, involving teacher and peer reports, are also needed to better understand the developmental and potentially causal dynamics underlying the relationships between bullying perpetration, victimization, and assertive behavior. Future studies may further examine assertive behavior in relation to different roles involved in bullying dynamics, including bully–victims and bystander roles such as assistants, reinforcers, outsiders, and defenders.

## 5. Conclusions

The present study identified several associations between bullying perpetration, victimization, and assertive behavior among Romanian primary school students aged 10–11 years. Positive associations were observed between bullying perpetration and victimization in both boys and girls. In addition, bullying perpetration and victimization were associated with assertive behavior and nonassertive responses (passive and aggressive behaviors), although most correlations were weak in magnitude. While the cross-sectional design does not allow causal interpretations, the findings highlight the complexity of bullying dynamics during late childhood. The results also suggest the potential relevance of school-based social and emotional learning and bullying prevention programs during this developmental stage.

## Figures and Tables

**Table 1:** The values of the Kolmogorov–Smirnov test.

Variable/Group	Boys	Girls
TB	0.000 *	0.000 *
PB	0.000 *	0.000 *
VB	0.000 *	0.000 *
SB	0.000 *	0.000 *
TV	0.000 *	0.000 *
PV	0.000 *	0.000 *
VV	0.000 *	0.000 *
SV	0.000 *	0.000 *
AsB	0.000 *	0.000 *
PaB	0.000 *	0.000 *
AgB	0.000 *	0.000 *

TB: Total Bullying; PB: Physical Bullying; VB: Verbal Bullying; SB: Social Bullying; TV: Total Victimization; PV: Physical Victimization; VV: Verbal Victimization; SV: Social Victimization; AsB: Assertive Behavior; PaB: Passive Behavior; AgB: Aggressive Behavior; * *p* < 0.001.

**Table 2:** Cronbach’s Alpha and Item-total statistics for the Romanian version of the CABS.

Item	Corrected Item-Total Correlation	Cronbach’s Alpha If Item Deleted
1	0.051	0.729
2	0.069	0.727
3	0.416	0.704
4	0.405	0.707
5	0.270	0.716
6	0.250	0.718
7	0.241	0.718
8	0.203	0.721
9	0.085	0.728
10	0.195	0.721
11	0.148	0.724
12	0.405	0.706
13	0.337	0.711
14	0.215	0.720
15	0.049	0.727
16	0.311	0.714
17	0.399	0.707
18	0.417	0.703
19	0.233	0.719
20	0.353	0.710
21	0.241	0.719
22	0.176	0.722
23	0.177	0.722
24	0.186	0.722
25	0.057	0.732
26	0.365	0.708
27	0.332	0.711
Cronbach’s		Alpha = 0.725

**Table 3:** Correlations between bullying perpetration, victimization, and assertive behavior among boys.

Variable	1	2	3	4	5	6	7	8	9	10
1. TB										
2. PB	0.695 **									
3. VB	0.839 **	0.484 **								
4. SB	0.725 **	0.354 **	0.384 **							
5. TV	0.485 **	0.358 **	0.423 **	0.418 **						
6. PV	0.498 **	0.381 **	0.399 **	0.372 **	0.765 **					
7. VV	0.432 **	0.345 **	0.388 **	0.370 **	0.870 **	0.527 **				
8. SV	0.395 **	0.238 **	0.303 **	0.397 **	0.771 **	0.462 **	0.555 **			
9. AsB	0.252 **	0.087	0.195 *	0.236 **	0.261 **	0.172 *	0.131	0.201 *		
10. PaB	−0.120	0.024	−0.112	−0.170 *	−0.210 **	−0.163 *	−0.031	−0.203 *	−0.738 **	
11. AgB	0.242 **	0.141	0.203 **	0.138	0.189 *	0.145	0.186 *	0.112	0.538 **	0.019

TB: Total Bullying; PB: Physical Bullying; VB: Verbal Bullying; SB: Social Bullying; TV: Total Victimization; PV: Physical Victimization; VV: Verbal Victimization; SV: Social Victimization; AsB: Assertive Behavior; PaB: Passive Behavior; AgB: Aggressive Behavior; ** *p* < 0.01; * *p* < 0.05.

**Table 4:** Correlations between bullying perpetration, victimization and assertive behavior among girls.

Variable	1	2	3	4	5	6	7	8	9	10
1. TB										
2. PB	0.551 **									
3. VB	0.819 **	0.377 **								
4. SB	0.844 **	0.335 **	0.465 **							
5. TV	0.530 **	0.241 **	0.415 **	0.491 **						
6. PV	0.393 **	0.264 **	0.268 **	0.364 **	0.725 **					
7. VV	0.479 **	0.225 **	0.403 **	0.389 **	0.846 **	0.543 **				
8. SV	0.431 **	0.163 *	0.328 **	0.437 **	0.735 **	0.397 **	0.470 **			
9. AsB	0.391 **	0.220 **	0.241 **	0.379 **	0.151	0.280 **	0.125	0.271 **		
10. PaB	−0.187 *	−0.083	−0.051	−0.213 **	−0.097	−0.169 *	−0.056	−0.164 *	−0.747 **	
11. AgB	0.350 **	0.167 *	0.313 **	0.311 **	0.089	0.143	0.063	0.157 *	0.518 **	0.057

TB: Total Bullying; PB: Physical Bullying; VB: Verbal Bullying; SB: Social Bullying; TV: Total Victimization; PV: Physical Victimization; VV: Verbal Victimization; SV: Social Victimization; AsB: Assertive Behavior; PaB: Passive Behavior; AgB: Aggressive Behavior; ** *p* < 0.01; * *p* < 0.05.

## Data Availability

The relevant data are reported in the study. The datasets are available upon request.
